# Beyond Blood Clotting: The Many Roles of Platelet-Derived Extracellular Vesicles

**DOI:** 10.3390/biomedicines12081850

**Published:** 2024-08-14

**Authors:** Barathan Muttiah, Sook Luan Ng, Yogeswaran Lokanathan, Min Hwei Ng, Jia Xian Law

**Affiliations:** 1 Department of Tissue Engineering and Regenerative Medicine, Faculty of Medicine, Universiti Kebangsaan Malaysia, Cheras, Kuala Lumpur 56000, Malaysia; lyoges@ppukm.ukm.edu.my (Y.L.); angela@ukm.edu.my (M.H.N.); 2 Department of Craniofacial Diagnostics and Biosciences, Faculty of Dentistry, Universiti Kebangsaan Malaysia, Jalan Raja Muda Abdul Aziz, Kuala Lumpur 50300, Malaysia; ngsookluan@ukm.edu.my

**Keywords:** platelet-derived extracellular vesicles (pEVs), hemostasis, angiogenesis, regenerative medicine, immune modulation

## Abstract

Platelet-derived extracellular vesicles (pEVs) are emerging as pivotal players in numerous physiological and pathological processes, extending beyond their traditional roles in hemostasis and thrombosis. As one of the most abundant vesicle types in human blood, pEVs transport a diverse array of bioactive molecules, including growth factors, cytokines, and clotting factors, facilitating crucial intercellular communication, immune regulation, and tissue healing. The unique ability of pEVs to traverse tissue barriers and their biocompatibility position them as promising candidates for targeted drug delivery and regenerative medicine applications. Recent studies have underscored their involvement in cancer progression, viral infections, wound healing, osteoarthritis, sepsis, cardiovascular diseases, rheumatoid arthritis, and atherothrombosis. For instance, pEVs promote tumor progression and metastasis, enhance tissue repair, and contribute to thrombo-inflammation in diseases such as COVID-19. Despite their potential, challenges remain, including the need for standardized isolation techniques and a comprehensive understanding of their mechanisms of action. Current research efforts are focused on leveraging pEVs for innovative anti-cancer treatments, advanced drug delivery systems, regenerative therapies, and as biomarkers for disease diagnosis and monitoring. This review highlights the necessity of overcoming technical hurdles, refining isolation methods, and establishing standardized protocols to fully unlock the therapeutic potential of pEVs. By understanding the diverse functions and applications of pEVs, we can advance their use in clinical settings, ultimately revolutionizing treatment strategies across various medical fields and improving patient outcomes.

## 1. Introduction 

Extracellular vesicles (EVs) have emerged as crucial mediators of intercellular communication, playing significant roles in various physiological and pathological processes [1]. Among them, platelet-derived extracellular vesicles (pEVs) are particularly noteworthy due to their abundance in human blood and their involvement in hemostasis, thrombosis, and beyond [2]. pEVs are also involved in a range of other physiological and pathological processes. They participate in intercellular communication by transferring proteins, lipids, and nucleic acids between cells, influencing various cellular functions and responses, which are crucial for cell communication, immune regulation, and tissue healing [3]. This review aims to elucidate the multifaceted roles of pEVs and their potential as liquid biomarkers, therapeutic agents, and diagnostic tools in biomedicine. Understanding the diverse functions and applications of pEVs can lead to breakthroughs in disease diagnosis, monitoring, and treatment, potentially revolutionizing patient care across various medical fields. Our hypothesis is that pEVs, due to their bioactive cargo and ability to facilitate intercellular communication, play pivotal roles in disease progression and tissue regeneration, making them valuable for diagnostics and therapeutics. The review is structured to first discuss the biogenesis and characterization of pEVs, providing foundational knowledge. It then explores the roles of pEVs in health and disease, emphasizing their significance in systemic signaling and pathogenesis. Following this, we highlight the therapeutic applications of pEVs in drug delivery and regenerative medicine, showcasing their potential to revolutionize patient care.

## 2. Platelets and Platelet-Derived Extracellular Vesicles (pEVs)

### 2.1. Platelets

Platelets, or thrombocytes, are essential fragments in blood responsible for hemostasis, preventing and controlling bleeding [4]. Derived from megakaryocytes in the bone marrow, platelets lack nuclei and appear as small disk-shaped fragments that circulate in the bloodstream with a physiological count of 150,000 to 450,000 platelets/µL and a lifespan of 8–10 days [5]. Platelets play a crucial role in both normal blood clotting and the formation of harmful blood clots. Typically, platelets are inactive while circulating in the blood, but they quickly respond to blood vessel damage by clumping together to stop bleeding [6]. Activation begins when von Willebrand Factor (vWF) and collagen, exposed during vascular injury, bind with receptors on platelets. This activation leads to the release of adenosine diphosphate (ADP) and the generation of thromboxane A2, further recruiting circulating platelets to form a hemostatic plug through the conversion of fibrinogen to fibrin [7]. All blood clots have different structures, reflecting the varying levels of platelet activation within them. Their primary function is to initiate blood clotting by adhering to exposed collagen in damaged blood vessels, forming a temporary plug to prevent excessive bleeding [8]. Key platelet receptors like glycoprotein (GP) Ib-IX-V and GP VI, and C-type lectin-like receptor 2 (CLEC-2) mediate platelet activation upon interaction with collagen or specific stimuli [9]. Platelet activation leads to shape change and release of granules containing bioactive molecules like clotting factors, growth factors, cytokines, and chemokines [10]. These molecules play crucial roles in clot formation, inflammation, wound healing, and immune response modulation [11]. Growth factors released from activated platelets, including platelet-derived growth factor (PDGF), epidermal growth factor (EGF), insulin-like growth factor-1 (IGF-1), transforming growth factor-beta (TGF-β), basic fibroblast growth factor (bFGF), and vascular endothelial growth factor (VEGF), promote cell proliferation, angiogenesis, and tissue repair during wound healing [12]. They play a crucial role in maintaining vascular integrity and regulating blood flow. Abnormalities in platelet function can lead to various bleeding or clotting disorders and cardiovascular diseases [13]. For instance, Bernard–Soulier Syndrome, a rare disorder caused by deficiencies in GPIb and GPV, results in impaired platelet adhesion and bleeding diathesis. Similarly, Glanzmann’s thrombasthenia, caused by a deficiency in GPIIb/IIIa, affects platelet aggregation and leads to bleeding complications [14,15]. A critical aspect of platelet function is the release of bioactive molecules through EVs. Understanding the intricate mechanisms of platelet activation and the release of bioactive molecules is essential for comprehending their role in health and disease. 

### 2.2. Biogenesis

**Figure 1:**
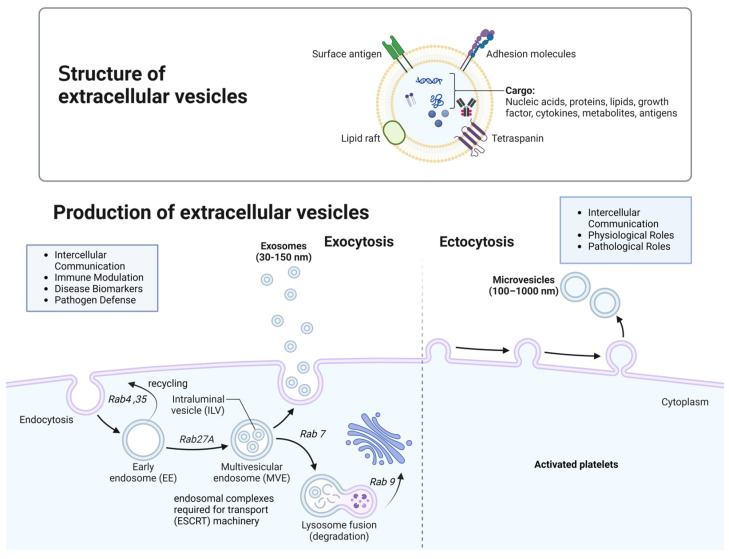
Biogenesis of extracellular vesicles. EVs are membrane-bound particles released by cells, classified into exosomes, microvesicles, and apoptotic bodies based on their origin and size. Exosomes form within multivesicular bodies and are released when these bodies fuse with the plasma membrane. Microvesicles bud directly from the plasma membrane, while apoptotic bodies result from cell fragmentation during apoptosis. EVs contain cellular lipids, proteins, and nucleic acids, reflecting their parent cell’s state and function. Understanding their biogenesis is essential for leveraging EVs in diagnostics and therapeutic applications.

Platelet activation triggers a cascade of events leading to the release of pEVs [16]. Common activators include calcium ionophores, thrombin, collagen, and other agonists that induce platelet activation and subsequent vesicle formation [17]. This process encompasses various cellular responses, including shape changes such as the extension of pseudopodia, and the secretion of granule contents such as ADP and growth factors. This process involves the externalization of phosphatidylserine (PS), creating a procoagulant surface and eventually cytoskeletal reorganization upon platelet activation, which is necessary for the formation of membrane blebs that will then bud off as pEVs [18]. The reorganization of the cytoskeleton and the exposure of PS lead to membrane blebbing and the shedding of microvesicles from the platelet surface. These microvesicles are a subset of pEVs, typically larger in size (100–1000 nm). Microvesicles are formed by direct budding from the plasma membrane of activated platelets, and carry platelet-specific surface markers. In addition to microvesicles, platelets can release exosomes, which are smaller EVs (30–150 nm) that originate from the endosomal compartment pathway [19]. The process starts with the formation of early endosomes (50 to 150 nm), which mature into late endosomes or multivesicular bodies (MVBs, 200 to 600 nm). Within MVBs, intraluminal vesicles (ILVs) are formed through inward budding of the endosomal membrane, encapsulating diverse cytoplasmic contents such as proteins, RNA (including mRNA and miRNA), lipids, and other molecules that are selectively packaged into these vesicles. The specific cargo can vary depending on the type of stimulus and the physiological or pathological context. Once formed, pEVs are released into the bloodstream, where they can interact with other cells and tissues, exerting their effects locally or at distant sites [18,19,20]. Early endosomes utilize several key pathways to transport and sort internalized cargo. They rapidly recycle receptors and membrane proteins back to the plasma membrane via Rab4 and Rab35, or more slowly through recycling endosomes involving Rab11. For degradation, early endosomes mature into late endosomes, acquiring Rab7, and eventually fuse with lysosomes for cargo breakdown. Additionally, early endosomes transport specific proteins and lipids to the trans-Golgi network via Rab9 [21]. They also form MVBs by creating ILVs through the endosomal complexes required for transport (ESCRT) machinery, which can either fuse with the plasma membrane to release exosomes or mature for degradation. These pathways ensure cellular homeostasis, regulate receptor levels, and manage the degradation of internalized materials [22]. Figure 1 shows the production of EVs.

### 2.3. Biochemistry of pEVs

pEVs have been a subject of intensive biomedical research since their initial discovery in the 1960s. Initially termed “platelet dust” or “platelet microparticles”, these small vesicles garnered attention for their role in platelet biology and hemostasis [23,24,25]. The pEVs are among the most abundant EVs in human blood, accounting for over half of all EVs in peripheral blood [26]. These pEVs are produced by activated platelets during physiological or pathological processes and influenced by triggers such as Ca^2+^ ionophore, ADP, thrombin, collagen, and epinephrine in determining their characteristics [24]. Protein levels in pEVs/exosomes were approximately 20 times lower than in platelets, suggesting that exosomes contribute minimally to the total protein content in activated platelet-rich plasma (PRP). pEVs contain a diverse range of bioactive molecules, contributing to various biological functions such as clotting, immune response, programmed cell death, growth factor signaling, and adhesion [25]. Key clotting factors in pEVs include the tissue factor (TF), factor Va (FVa), and FVIII, which play a role in the coagulation cascade [27]. Adhesion molecules like CD41/61 (GPIIb/IIIa), CD31, and CD62P (P-selectin) are crucial for platelet aggregation and adhesion [28]. Enzymes such as heparanase and protein disulfide isomerase (PDI) are also present, along with bioactive lipids like arachidonic acid (AA) and thromboxane A2 (TXA2) [28]. pEVs contain growth factors including TGF-β1, PDGF, and VEGF, which contribute to tissue repair and regeneration [29]. In terms of the immune response, chemokines like CCL5 and CCL23, cytokines such as interleukin-1 beta (IL-1β), and complement components (C5b-9) play a role in modulating inflammation and immunity [30]. Additionally, pEVs carry components related to programmed cell death, such as PS, caspase-3, caspase-9, and damage-associated molecular patterns (DAMPs), reflecting their role in apoptotic pathways [31]. These vesicles play a key role in intercellular communication, signaling, and immune responses, and their unique ability to cross tissue barriers makes them valuable for potential therapeutic applications [23]. pEVs are often identified by the presence of specific proteins, with the most common being tetraspanins (like CD9 and CD63), tumor susceptibility gene 101 (TSG101), programmed cell death 6-interacting protein (PDCD6IP or ALIX), CD41 (GPIIb), CD42a (GPIX), CD62P (P-selectin), CD31 (PECAM-1), and LAMP-1 (CD107a), which indicate their source and functional abilities. These markers also reveal pEVs’ role in platelet aggregation, adhesion, and interactions with leukocytes and endothelial cells [32,33]. 

PRP is classified into four types based on leukocyte and fibrin contents: leukocyte-rich PRP (L-PRP), leukocyte-reduced PRP (P-PRP), leukocyte-platelet-rich fibrin, and pure platelet-rich fibrin. Activated PRP is known to be rich in PRP-EVs [34]. When PRP-EVs were initially found, they were believed to carry clotting substances, carrying out roles akin to platelets [35]. Advancements in research have unveiled more functions of PRP-EVs, such as involvement in blood clotting, maintaining blood vessel health, controlling immune responses, and managing inflammation. The unique features of PRP-EVs, such as low immunogenicity, protection from degradation, facilitation of intercellular communication, and ability to cross biological barriers, contribute to their therapeutic efficacy [36]. PRP-EVs were formed intracellularly within bilayer lipid bodies and have common surface markers, such as CD9, CD63, CD81, and HSP101 [34]. This type of EV is also rich in bioactive molecules like PDGF, TGF-β, VEGF, IL-1β, and tumor necrosis factor-alpha (TNF-α) [35,36]. PRP-EVs modulate various signaling pathways, including Wnt/β-catenin for cell proliferation and differentiation, AKT/ERK for cell survival and growth, PI3K/Akt for cellular metabolism and apoptosis, and Keap1-Nrf2 for regulating oxidative stress and inflammation. They are applied across diverse medical fields such as orthopedics, dermatology, and wound healing, where clinical trials have confirmed their effectiveness in promoting tissue repair and reducing inflammation. PRP-EVs offer notable advantages over other regenerative therapies, including a reduced risk of immune rejection and easier administration, while providing a concentrated source of regenerative molecules with fewer complications compared to whole PRP preparations [37,38].

Both pEVs and PRP-EVs offer significant potential for therapeutic applications, with research focusing on their roles in regenerative medicine, tissue repair, and other clinical uses due to their safety and efficacy profile [38]. Table 1 provides a concise comparison between PRP-EVs and pEVs regarding their origin, composition, and applications. While PRP-EVs are known for their roles in tissue regeneration and healing, this review will exclusively focus on pEVs. We will explore their origins, characteristics, and roles in various biological processes, emphasizing their distinct contributions and significant therapeutic potential due to their abundance in human blood. 

### 2.4. Isolation of pEVs

**Figure 2:**
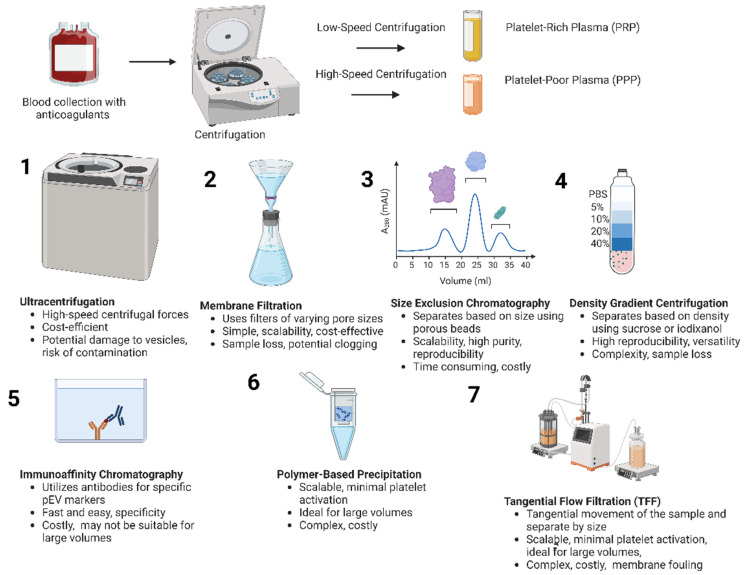
Isolation of platelet-derived extracellular vesicles (pEVs). Various methods are used to isolate pEVs, each with advantages and limitations. Ultracentrifugation is cost-efficient but may damage vesicles, while membrane filtration is expensive. Gel filtration separates based on size, density gradient centrifugation offers reproducibility, and immunoaffinity chromatography is rapid but costly. Polymer-based precipitation is simple but may co-precipitate other molecules, and tangential flow filtration is ideal for clinical applications but complex and costly. The choice of method depends on factors like sample volume, purity requirements, cost, and reproducibility.

This method allows for the production of different types of pEVs depending on the activation agent used, providing versatility in pEV characterization. However, some activation methods may lead to lower procoagulant activity and can be costly [24]. Isolation of pEVs involves several key steps, beginning with careful blood collection using anticoagulants like sodium citrate or ACD to prevent clotting. The blood is then centrifuged at low speed to obtain PRP, which is further processed by higher-speed centrifugation to remove platelets and yield platelet-poor plasma (PPP) [39,40]. This is followed by ultracentrifugation, the traditional method for isolating pEVs, which relies on high-speed centrifugal forces to separate particles by density [41]; while centrifugation is cost-efficient and can yield pure preparations, it may result in low reproducibility and potentially damage exosomes due to high centrifugal forces as well as carries risks of contamination with lipoproteins and other impurities [42]. Alternative methods include membrane filtration, which employs a series of filters with different pore sizes to isolate pEVs. The process typically begins with larger pore sizes to remove cells and larger particles, then progressively uses smaller pore sizes to isolate the desired pEV population [43]. Gel filtration (e.g., size exclusion chromatography) separates vesicles based on size using columns filled with porous beads [44]. This method utilizes columns packed with porous beads of a specific material, typically cross-linked agarose or dextran [44]. Density gradient centrifugation separates vesicles based on their density using materials like sucrose or iodixanol. This provides a more refined isolation process with high reproducibility and preserved vesicle integrity [45]. These methods are generally straightforward, allowing for the processing of multiple samples simultaneously, but they can be expensive and require specialized equipment. Immunoaffinity chromatography and immuno-bead capturing leverage antibodies specific to pEV markers to provide fast and easy isolation with significant enrichment, but these techniques may not be suitable for large-volume samples and can incur higher costs [46]. Iodixanol density gradient and immuno-bead capturing offer pure preparations without viral particles but might result in sample loss and difficulty in separating large particles with similar sedimentation rates [47]. Polymer-based precipitation (e.g., ExoQuick™) is a method that offers a simpler and faster alternative to ultracentrifugation. In this approach, a polymer solution is added to the biological fluid containing EVs, typically platelet-poor plasma for pEV isolation [48]. While this method is quicker and requires less specialized equipment than ultracentrifugation, it may co-precipitate other molecules and proteins, potentially affecting the purity of the isolated EVs. Therefore, additional purification steps may be necessary depending on the intended downstream applications [49]. Tangential flow filtration (TFF) offers cleaner and more scalable options for pEV isolation utilizing a porous membrane for large-scale purification. This method is particularly well-suited for clinical use due to its minimal platelet activation, a crucial factor in maintaining the native state of pEVs. TFF’s ability to process large volumes efficiently while preserving pEV integrity makes it ideal for industrial or clinical applications where higher purity and larger sample volumes are required. The choice between these methods ultimately depends on the specific needs of the research or clinical project, considering factors such as sample volume, required purity, cost, reproducibility, and intended downstream applications [50,51]. Figure 2 shows the various methods to isolate pEVs. 

## 3. Biological Functions of pEVs 

### 3.1. pEVs in Cancer

pEVs are gaining attention for their roles in tumor growth and metastasis, with increased levels observed in patients with various cancers like glioblastoma, gastric, lung, and skin cancer [52]. This has sparked interest in their potential as diagnostic markers and as early indicators of disease progression [53]. It is established that platelets facilitate cancer metastasis, and recent research explores pEVs’ contribution to cancerogenesis [54]. As cell-to-cell messengers, pEVs can transfer bioactive molecules to cancer cells, cancer-associated fibroblasts, endothelial cells, and tumor-associated macrophages in the tumor microenvironment (TME), thereby impacting both tumor cell behavior and the surrounding environment [55].

pEVs are found to act as facilitators for the formation of new blood vessels, a critical step for cancer progression known as angiogenesis [56]. Happonen et al. revealed that endothelial cells, which line blood vessel walls, engulf PS-positive pEVs through a process called phagocytosis. This engulfing appears to involve Axl, a tyrosine kinase receptor, and its partner protein Gas6 [57]. This process provides insights into how pEVs might modulate vascular health and disease. Furthermore, targeting the Axl-Gas6 pathway could offer therapeutic potential for controlling pEV-mediated effects in pathological conditions such as thrombosis, atherosclerosis, and inflammation [58]. In addition, studies have reported the transfer of molecules like miR-126, VEGF, PDGF, and FGF to endothelial cells through pEVs to stimulate angiogenesis [59,60,61]. This specific interaction hints at a potential pathway for pEVs to influence angiogenesis. Further evidence comes from Żmigrodzka et al. and Liang et al., who observed a significant rise in mRNA expression for key pro-angiogenic factors like matrix metalloproteinase (MMP)-9, IL-8, VEGF, miR-223, and scatter factor in lung cancer cell lines upon exposure to pEVs [52,62]. MMP-1 is also transferred from pEVs to the TME to bolster angiogenesis [63]. These findings suggest that pEVs might contribute to creating an environment conducive to new blood vessel formation, ultimately supporting tumor growth. Anene et al. provided evidence for pEV-mediated manipulation of angiogenesis using human umbilical vein endothelial cells (HUVECs). They showed that pEV-derived miR-let-7a targets thrombospondin-1 (THBS-1) in HUVECs, lowering its production and promoting blood vessel formation [64]. This shift disrupts the delicate balance between pro- and anti-angiogenic factors, triggering the “angiogenic switch” crucial for tumor expansion [65].

pEVs are not solely focused on blood vessel growth to stimulate cancer progression. They can also worsen metastasis by promoting epithelial-to-mesenchymal transition (EMT). This process allows epithelial cancer cells, typically confined in a tissue layer, to transform into more mobile mesenchymal cells, increasing their invasive potential [66]. pEVs can transport microRNAs (miRNAs) like miR-939, promoting the aggressiveness of ovarian cancer cells. MiR-939 modulates gene expression by targeting mRNAs. In ovarian cancer, miR-939 regulates pathways crucial for cancer progression, enhancing cell proliferation, migration, invasion, and resistance to apoptosis [67]. miR-939 targets tumor suppressor genes and extracellular matrix components, promoting invasiveness. pEVs deliver miR-939 to distant sites, facilitating metastasis by protecting it from degradation. This insight into ovarian cancer metastasis suggests new therapeutic strategies targeting pEV pathways or specific miRNAs for treatment [68]. Studies have shown that pEVs can deliver miRNAs like miR-939, which enhance the aggressiveness of ovarian cancer cells, highlighting another way pEVs contribute to cancer’s spread [69]. Additionally, pEVs have been found to enhance invasion of prostate cancer cells via the upregulation of MMP-2 expression, which is partially driven by protein kinase C (PKC) and tyrosine phosphorylation pathways [70]. In a separate study, the authors found that pEVs enhance the migration of breast cancer cells through the partial remodeling of calcium handling machinery to modulate cancer cell motility. The cancer cell pretreated with pEVs demonstrated higher calcium ion release from the endoplasmic reticulum, triggering store-operated Ca^2+^ entry (SOCE), a calcium entry pathway on the plasma membrane. This potentiates the cancer cell’s serum-induced migration through the activation of p38 MAPK and MLC2 signaling pathways [71].

pEVs also actively modulate the immune response, potentially favoring cancer progression. For instance, pEVs can transfer miR-183 to natural killer (NK) cells, dampening their ability to kill cancer cells [72]. Furthermore, pEVs can reprogram macrophages in the TME through the transfer of miR-126-3p, making them more likely to engulf and clear other immune cells, potentially suppressing the overall anti-tumor immune response [73]. Selective polarization of macrophages to the M2 phenotype by pEVs also stimulated monocyte chemotaxis, adhesion, and differentiation into a resident M2 subset, promoting phagocytosis while inhibiting cell proliferation by creating an immunosuppressive TME [74]. In addition, pEVs have been observed to reduce Jurkat cell mobility, which might contribute to immune evasion by decreasing leukocyte invasiveness [74]. These findings highlight the multifaceted role of pEVs in shaping the TME and influencing cancer development. Contursi et al. found that pEVs from colorectal cancer (CRC) patients increase the EMT markers, cyclooxygenase (COX)-2 (PTGS2) expression, and thromboxane (TX) B2 production in CRC cell lines. These observations suggests that pEVs could drive prometastatic and prothrombotic behaviors in cancer cells, indicating potential treatment targets [75].

Despite their potential to promote cancer, some studies suggest that pEVs might also be a double-edged sword. They can deliver miR-24 into cancer cells, triggering apoptosis and hindering tumor development by inhibiting new blood vessel growth through the transfer of specific miRNAs like miR-223 [73]. This suggests that pEVs might have potential anti-cancer properties that warrant further investigation. Along with this, researchers compared two methods for creating drug-loaded pEVs for potential cancer therapy. They found that loading the drug doxorubicin (DOX) directly into isolated pEVs was more efficient than first loading it into platelets and then isolating the pEVs. These DOX-loaded pEVs effectively killed cancer cells, with a particular breast cancer cell line (MDA-MB-231) showing the most sensitivity to this treatment. This suggests that directly loading drugs into pEVs might offer a promising strategy for targeted cancer therapy [14,76]. Gasperi et al. showed that pEVs with miR-126 and miR-223 increased the sensitivity of BT549 cells to cisplatin chemotherapy [77]. miR-126 and miR-223 play important roles in enhancing the effectiveness of chemotherapy by targeting genes that promote drug resistance in cancer cells. miR-126 suppresses angiogenesis and cell survival pathways, while miR-223 regulates apoptosis and cell cycle progression [78]. A recent study identified AKT2 kinase as a direct target of miR-126-3p, a key variant of miR-126. Through bioinformatic analysis and luciferase assays, researchers found that both ectopic expression and pEV-mediated delivery of miR-126-3p suppressed AKT2 expression, reducing proliferation and invasion in triple-negative (BT549) and luminal A (MCF-7) breast cancer subtypes [79].

### 3.2. pEVs in Viral Infection 

The systemic inflammatory response triggered by SARS-CoV-2 infection involves various components, including platelets and the coagulation system [80,81]. Platelets, besides their primary role in hemostasis, also contribute to the immunoinflammatory response against infection [82]. SARS-CoV-2 infection triggers a complex interplay between platelets and inflammation [83]. COVID-19 patients show increased levels of pEVs compared to healthy individuals, potentially serving as diagnostic markers. These elevated pEVs, particularly platelet small EVs (sEVs), might be linked to blood clotting issues observed in severe cases. The virus may also interact with pEVs to worsen inflammation through the VWF-ADAMTS13 axis [84]. This suggests the relevance of pEVs in SARS-CoV-2 infection and their potential as diagnostic biomarkers, supported by their strong association with infection and good diagnostic performance. Circulating EVs contribute to thrombo-inflammation by stimulating pro-inflammatory endothelial cytokines like IL-8, IL-6, and MCP-1, which can lead to endothelial damage and senescence [85]. pEVs were also elevated in COVID-19 patients compared to healthy individuals, despite no change in platelet count, indicating that they could serve as biomarkers for SARS-CoV-2 infection. These EVs contain procoagulant and immune mediators that promote platelet aggregation and coagulation pathways [86].

Interestingly, similar interactions between viruses and pEVs have been observed in dengue fever. Dengue virus activates platelets, leading to pEV secretion that promotes neutrophil activity and inflammation [87]. Additionally, platelet-derived exosomes (pEXOs) in dengue patients disrupt blood vessel integrity and increase inflammation, potentially explaining vascular leakage and thrombocytopenia (low platelet count) observed in severe cases. Platelet activation correlated with disease severity, and dengue viral activation led to the release of pEXOs expressing CD63 and CD9 [88]. These findings highlight the multifaceted role of pEVs in viral infections and warrant further investigation into their potential as diagnostic tools and therapeutic targets.

### 3.3. pEVs in Wound Healing 

A clinical trial evaluating pEVs purified via Large-scale Exosome Affinity Purification (LEAP) demonstrated their scalability and efficiency for clinical-grade EV purification. While focusing on safety in healthy individuals, the trial showed enhanced cell functions without significant adverse events reported. However, wound closure time did not significantly differ between treated and untreated groups, likely due to study design limitations. Future trials should consider chronic wound patient dynamics and sustained pEV delivery via hydrogels [89]. Studies using hydrogels loaded with pEVs have shown remarkable progress. For example, gelatin-alginate hydrogel (GelAlg) loaded with reduced graphene oxide (rGO) (GelAlg@rGO) containing pEVs promoted wound healing in diabetic rats by reactive oxygen species (ROS) scavenging abilities and immune modulation, facilitating macrophage transition to the anti-inflammatory M2 phenotype [90]. Additionally, a resveratrol-pEV incorporated gelatin methacrylate (GelMA) and silk fibroin glycidyl methacrylate (GelMA/SFMA) composite hydrogel exhibited similar benefits in diabetic mice, whereby it reduced pro-inflammatory factors’ (TNF-α and iNOS) expression while boosting anti-inflammatory factors (TGF-β1 and Arg-1), further highlighting the potential of pEVs for chronic wound treatment [91]. These findings suggest that pEV-loaded hydrogels represent a promising strategy for accelerating wound closure and improving healing outcomes, especially in diabetic patients.

### 3.4. pEVs in Osteoarthritis

Osteoarthritis (OA) is a chronic joint disease that predominantly affects the elderly population, causing pain, stiffness, and reduced mobility [92]. pEXOs have shown promise in alleviating OA symptoms by promoting chondrocyte function and cartilage regeneration. In a recent study, pEXOs were isolated from human platelets and demonstrated significant efficacy in enhancing chondrocyte proliferation and migration under inflammatory conditions. Moreover, intra-articular injection of pEXOs in an OA mouse model led to cartilage regeneration and slowed disease progression. Transcriptomic analysis revealed the underlying molecular mechanisms, highlighting the potential of pEXOs in OA therapy [93]. Comparative studies have shown that pEVs outperform mesenchymal stem cell-derived EVs in promoting chondrocyte proliferation and mitigating cartilage degradation in OA models. Notably, pEVs exhibited superior cellular uptake and therapeutic effects compared to cellular exosomes, indicating their potential as a regenerative therapy for OA. These findings underscore the importance of further clinical investigation to validate the efficacy and safety of pEVs in treating OA [94].

### 3.5. pEVs in Sepsis 

Urosepsis, a severe complication of genitourinary infections, carries high mortality rates and often leads to acute kidney injury (AKI), worsening patient outcomes [95]. pEVs offer promise as biomarkers [96], showing a stronger correlation with AKI severity than traditional markers like blood urea nitrogen (BUN), serum creatinine (SCr), and neutrophil gelatinase-associated lipocalin (NGAL), enabling earlier AKI detection [97].

Lu et al. have shown that pEVs contribute to inflammation, apoptosis, and oxidative stress in renal tubular epithelial cells (RTECs). EVs from platelets activated by lipopolysaccharide (LPS), a bacterial toxin, raise SCr and BUN levels, indicating impaired kidney function in sepsis. The damage involves ARF6, a molecule enriched in these EVs, with activation triggered by the TLR4/MyD88 pathway. This suggests that targeting ARF6 could offer a therapeutic approach to mitigate sepsis-related kidney injury [98].

Their impacts on the inflammatory response, neutrophil extracellular trap (NET) formation, and endothelial dysfunction were studied recently. Utilizing a sepsis rat model, investigators found that pEVs exacerbated NET formation, endothelial dysfunction, and the inflammatory response compared to the control group [99]. Yet, the precise mechanisms underlying pEVs’ role in regulating inflammation and biomarkers in sepsis remain elusive, emphasizing the necessity for further investigation to elucidate the factors influencing sepsis progression and identify potential therapeutic targets [100].

### 3.6. pEVs and Cardiovascular Diseases

pEVs are crucial in platelet activation and superoxide generation, mainly through the enzyme Nox-1. When platelets activate, Nox-1 transfers to pEVs, where it drives superoxide production. This superoxide generation can activate nearby platelets, potentially leading to thrombo-inflammatory conditions. Inhibiting Nox-1 in pEVs could reduce platelet activation and control such inflammatory responses, indicating a potential therapeutic approach [101]. Another study found that patients with permanent atrial fibrillation (AF) have higher levels of PS-carrying pEVs, indicating a pro-coagulant state. In contrast, patients with intermittent AF, who have periods of normal sinus rhythm, exhibit lower levels of these vesicles, suggesting that restoring sinus rhythm might lower the risk of stroke and thromboembolism by reducing pro-coagulant vesicles [102].

Researchers also uncovered a new thrombo-inflammatory pathway involving pEVs in monocyte recruitment, where pEVs transfer GPIbα to monocytes, enabling them to adhere to von Willebrand factor (VWF) on the vessel wall. This leads to monocyte migration to inflamed areas and may contribute to chronic inflammatory diseases and trauma complications [103]. A vicious cycle in sickle cell disease (SCD) involves platelets containing more inflammation-promoting IL-1β, with bacterial exposure increasing IL-1β packaging within EVs. These pEVs can cause platelet–neutrophil clumping, leading to pulmonary vaso-occlusion. However, blocking IL-1β or its activating enzyme, caspase-1, prevents lung blockages, suggesting that targeting this pathway could offer therapeutic benefits for SCD-related lung complications [104]. 

### 3.7. pEVs in Rheumatoid Arthritis

A recent study has found that pEVs play a role in rheumatoid arthritis (RA) by entering the lymphatic system and traveling to other parts of the body beyond the inflamed joints, whereby pEVs have been detected in the synovial fluid of RA patients, indicating they have already infiltrated the joints. pEVs transport platelet components, like certain inflammatory molecules, to other areas, contributing to the spread of inflammatory signals in RA [105]. For example, pEVs can transport miRNAs and cytokines, which have the potential to affect immune responses, contributing to disease progression and possibly adding to the cardiovascular risks linked with RA. Additionally, pEVs have been observed in the lymphatic system of mouse models of atherosclerosis, suggesting a role in other vascular inflammatory conditions as well [106].

### 3.8. pEV in Atherothrombosis

**Figure 3:**
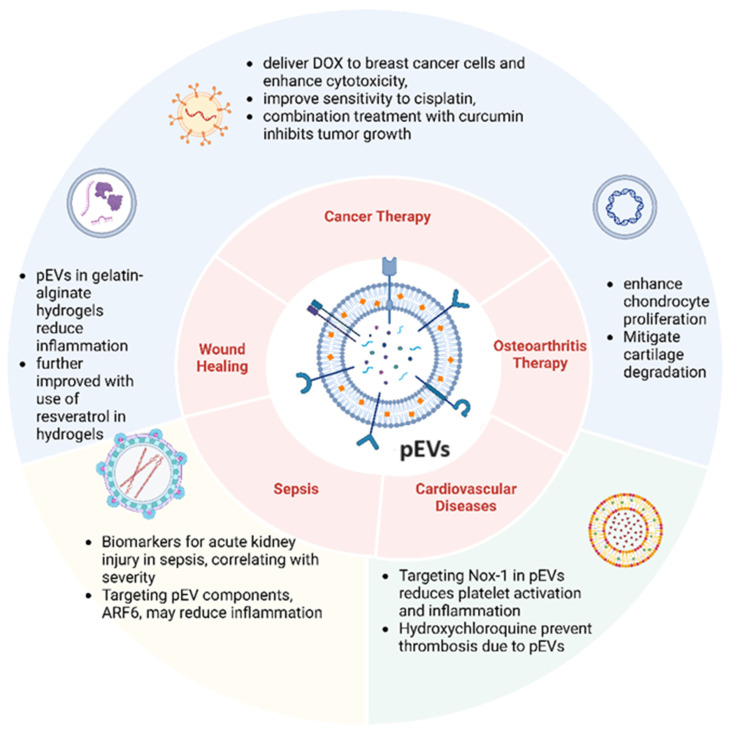
Therapeutic potential of pEVs. pEVs have therapeutic potential in cancer, wound healing, osteoarthritis, sepsis, and cardiovascular diseases through various mechanisms.

Thrombin-induced platelet activation leads to the release of EVs carrying stress-related proteins like GRP78 and cytoskeleton-related proteins essential for cell adhesion, signal transduction, and thrombus formation [107]. This activation also modifies the expression of proteins involved in kinase activity and Wnt/β-catenin signaling, amplifying platelet activation. Moreover, changes in oxidoreductases like PDIA1 and PDIA3 upon platelet activation suggest a redox remodeling state on the platelet surface, facilitating the activation of key proteins such as αIIbβ3-integrin. This indicates that targeting proteins carried by thrombin-induced pEVs could offer a therapeutic approach against atherothrombosis progression [107]. In a related study, trauma-derived pEVs were found to have a dual role: promoting hemostasis post-injury while potentially driving thrombosis later. Hydroxychloroquine (HCQ) treatment can reduce the release and effects of trauma-derived pEVs, suggesting its potential use as an antiplatelet and antithrombotic prophylaxis for deep vein thrombosis (DVT). Further studies are needed to clarify the mechanisms behind EV-induced platelet activation and to explore HCQ’s role in reducing thrombotic risks post-trauma [108]. Table 2 displays a summary of the multifaceted roles of pEVs in various physiological and pathological processes. Figure 3 illustrates the therapeutic potential of pEVs. 

## 4. Perspectives

Future research directions for pEVs cover a broad spectrum, from cancer therapy to cardiovascular health, since pEVs offer a myriad of advantages in biomedical applications [14]. Laden with bioactive molecules, they stimulate tissue regeneration and repair, making them valuable in wound healing and tissue engineering. Their angiogenic properties foster vascularization in ischemic tissues, while their immunomodulatory effects hold promise for managing autoimmune diseases. With a stable structure and minimally invasive delivery routes, pEVs present a safe and effective therapeutic option with broad clinical potential [15,16,17]. In addition, the unique characteristics of pEVs, such as their ability to circulate for extended periods and their capacity to penetrate tissue barriers like the blood–brain barrier, make them attractive candidates for regenerative medicine and drug delivery since pEVs also possess key surface markers, like CD41 and CD62P, that mediate targeting to inflammation and tumor sites [109]. Given their stability, low immunogenicity, and capacity for tissue penetration, pEVs are increasingly being explored for regenerative medicine and drug delivery applications [39]. Notably, pEVs can be loaded with drugs through several methods, such as incubation, sonication, electroporation, extrusion, dialysis, freeze–thaw, and transfection, to name a few [110]. One of their key advantages is the ability to carry both hydrophobic and hydrophilic drugs, enabling them to deliver therapeutic agents to specific targets, including inflamed tissues or tumor sites [111]. For example, certain studies suggest that pEVs containing miR-24 can trigger tumor cell apoptosis, while others demonstrate their ability to deliver drugs like DOX and bortezomib to cancer cells, enhancing the efficacy of treatment [74]. Future investigations might focus on harnessing this targeted delivery system to improve cancer treatment outcomes, while also understanding how pEVs influence the TME, immune modulation, and metastasis.

Beyond cancer therapy, pEVs also play significant roles in immuno-inflammation, thrombosis, and cardiology. They can act as extracellular traps for neutrophils, contribute to inflammation by recruiting leukocytes, and facilitate interactions between monocytes and endothelial cells [100]. pEVs’ inherent affinity for inflammation sites, such as atherosclerotic plaques, allows them to serve as drug delivery vehicles for anti-inflammatory agents. Ma et al. demonstrated that pEVs were engineered to target inflammatory sites along with anti-inflammatory drugs like [5-(p-fluorophenyl)-2-ureido]thiophene-3-carboxamide (TPCA-1), offering a novel drug delivery system for treating pneumonia. Beyond pneumonia, pEVs show potential in targeting various inflammation sites, including atherosclerotic plaques and RA, suggesting their broader application in disease treatment [111].

In thrombosis, pEVs exhibit high procoagulant activity, leading to clot formation and suggesting their use as biomarkers for arterial thrombosis. In cardiology, pEVs have demonstrated pro- and anti-angiogenic potential, promoting cardiac regeneration and guiding living cells towards injured tissues [112,113]. Similarly, pEVs can mediate platelet activation and thrombo-inflammatory conditions, suggesting that targeting specific molecules like Nox-1 could mitigate these effects [102]. For sepsis, studies are exploring how pEVs contribute to inflammation and renal injury, with an emphasis on potential therapeutic targets like ARF6. pEVs derived from healthy humans have shown significantly decreased myocarditis, lowered infiltration of macrophages, T cells, and mast cells, and improved cardiac function. Additionally, pEV treatment reduced fibrosis and remodeling markers, and its miRNA content revealed the ability to modulate pathways involved in viral replication, TLR4 signaling, and T-cell activation. These findings suggest that pEVs could serve as a promising therapy for reducing inflammation and improving outcomes in viral myocarditis patients [114,115,116]. pEVs can boost the therapeutic potential of adipose-derived mesenchymal stem cells (ADSCs) used to treat ischemic diseases. A study found that in a mouse model of hindlimb ischemia, ADSCs treated with pEVs showed improved vascularization, tissue regeneration, and blood flow compared to controls. This suggests that pEVs can enhance the angiogenic potential of ADSCs, offering a promising strategy to improve stem cell therapies for ischemic conditions [117]. These diverse applications indicate that pEVs can offer both a diagnostic tool and a therapeutic target, underscoring the need for further studies to understand their full range of functions and how they can be leveraged to develop new treatments. pEVs continue to gain attention in the fields of regenerative medicine such as bone, muscle, cartilage, and skin thanks to carrying growth factors like PDGF, FGF2, TGF-β, and VEGF. These factors stimulate cell proliferation, angiogenesis, and tissue repair, making pEVs ideal candidates for wound healing and tissue regeneration. More research is required to understand their full potential and overcome technical challenges [80,118,119]. Although pEVs hold significant therapeutic potential, further research is needed to standardize their isolation, storage, and preparation methods to fully realize their clinical applications and ensure safety and efficacy in regenerative medicine and beyond. This includes optimizing preparation methods, ensuring safety and quality control, and exploring new strategies to improve drug-loading efficiency. Ultimately, pEVs represent a promising platform for innovative therapies, with the potential to revolutionize treatment approaches across a range of diseases.

## 5. Conclusions

pEVs play a multifaceted role in health and disease. These tiny vesicles, released from activated platelets, contain a diverse array of bioactive molecules that allow them to communicate with other cells, influencing a wide range of biological activities such as intercellular communication, and impacting processes like coagulation, inflammation, immune response modulation, and tissue regeneration. pEVs contribute to various diseases, including cancer, sepsis, OA, cardiovascular diseases, SCD, RA and atherothrombosis. Their functions range from promoting tumor growth and angiogenesis to exacerbating inflammation and aiding in disease progression. 

Despite their considerable potential, pEVs face challenges related to technical limitations, safety concerns, and variability in preparation methods. Future research should aim to optimize pEV isolation techniques, explore innovative drug-loading methods, and conduct rigorous clinical trials to validate their therapeutic benefits. In conclusion, pEVs offer a compelling platform for innovative therapies across various medical fields. Their unique properties and broad applications suggest they could revolutionize treatment approaches and improve patient outcomes. Continued exploration and research will be crucial to fully realize the potential of pEVs and translate their benefits into clinical practice.

## Figures and Tables

**Table 1:** Comparison between platelet-rich plasma extracellular vesicles (PRP-EVs) and platelet-derived extracellular vesicles (pEVs).

Feature	Platelet-Rich Plasma Extracellular Vesicles (PRP-EVs)	Platelet-Derived Extracellular Vesicles (pEVs)
Origin	Derived from platelet-rich plasma obtained by centrifugation of whole blood [34]	Produced by activated platelets during physiological or pathological processes [23]
Formation	Formed intracellularly within bilayer lipid bodies [34]	Released from platelets in response to various triggers such as Ca^2+^ ionophore, ADP, thrombin, collagen, and epinephrine [24]
Common Surface Markers	CD9, CD63, CD81, HSP101 [34]	CD9, CD63, TSG101, PDCD6IP (ALIX) [32,33]
Key Clotting Factors	-	Tissue factor (TF), factor Va (FVa), factor VIII (FVIII) [27]
Adhesion Molecules	CD31, CD41, CD42a, CD61, CD62p, CD63 and PS, GPIbα [34]	CD41/61 (GPIIb/IIIa), CD31, CD62P (P-selectin) [28]
Enzymes	Thrombin, matrix metalloproteinases, alkaline phosphatase [35]	Heparanase, protein disulfide isomerase (PDI), NADPH oxidase 1, glutathione peroxidase, alkaline phosphatase, phospholipase A2 [28]
Bioactive Lipids	Ceramides, phosphatidylserine [36]	Lysophospholipids (LPLs), glycerolipids (GLs), phospholipids (PLs), sphingolipids (SLs), L-carnitine, arachidonic acid (AA), thromboxane A2 (TXA2) [28]
Growth Factors	PDGF, TGF-β, VEGF, IL-1β, TNF-α [36]	TGF-β1, PDGF, VEGF [29]
Chemokines	PF4, PPBP, CCL5 (RANTES) [36]	CXCR1, PF4, PPBP [30]
Immune Response Molecules	IL-1β, IL-6, IL-8, IL-10, CXCL1, CXCL2, CXCL8 [36]	CCL5, CCL23, IL-1β, complement components (C5b-9) [30]
Components Related to Apoptosis	Caspase, Bcl-2 family, Apaf-1 [31]	Phosphatidylserine, caspase-3, caspase-9, DAMPs [31]
Signaling Pathways	Wnt/β-catenin, AKT/ERK, PI3K/Akt, Keap1-Nrf2 [36]	TGF-beta, NF-κB, MAPK/ERK, PI3K/Akt, Wnt/β-catenin, Notch [36]
Functions	Supports tissue regeneration and healing Reduces inflammation, apoptosis, oxidative stress, and senescence [36]	Clotting, immune response, programmed cell death, growth factor signaling, adhesion [25]
Applications	Tissue regeneration, healing [37,38]	Diagnostics, therapeutic applications in cardiovascular disorders, cancer, regenerative medicine [37,38]

**Table 2:** The multifaceted roles of pEVs.

Disease/Condition	Role of pEVs	Potential Therapeutic Application	References
Cancer	pEVs contribute to tumor growth, metastasis, angiogenesis, and epithelial-to-mesenchymal transition, and they transfer bioactive molecules to cancer cells and the tumor microenvironment.	Targeting pEV pathways or specific miRNAs for treatment; drug-loaded pEVs for targeted therapy.	[52,53,54,55,56,57,58,59,60,61,62,63,64,65,66,67,68,69,70,71]
Viral Infection (COVID-19)	Elevated pEV levels observed in patients, linked to blood clotting issues and inflammation.	Potential as diagnostic biomarkers; targeting pEVs to mitigate inflammatory and thrombotic responses.	[81,82,83,84,85,86]
Viral Infection (Dengue)	pEVs promote neutrophil activity and inflammation, disrupt blood vessel integrity, and increase vascular leakage.	Investigating pEVs as diagnostic tools and therapeutic targets to manage inflammation and vascular damage.	[87,88,89]
Wound Healing	pEV-loaded hydrogels promote wound healing by scavenging reactive oxygen species and modulating immune responses.	Developing pEV-loaded hydrogels for chronic wound treatment, particularly in diabetic patients.	[90,91,92]
Osteoarthritis (OA)	pEVs enhance chondrocyte function and cartilage regeneration, alleviate symptoms by promoting chondrocyte proliferation and migration.	Developing pEV-based therapies for cartilage regeneration and OA treatment.	[93,94,95]
Sepsis	pEVs contribute to inflammation, apoptosis, oxidative stress, and endothelial dysfunction in sepsis, leading to kidney injury and worsened outcomes.	Targeting pEVs to mitigate sepsis-related kidney injury and inflammatory responses.	[96,97,98,99,100,101]
Cardiovascular Diseases	pEVs drive platelet activation, superoxide generation, monocyte recruitment, and thrombo-inflammatory conditions.	Inhibiting pEV pathways to reduce platelet activation and control inflammatory responses.	[102,103,104,105]
Rheumatoid Arthritis (RA)	pEVs spread inflammatory signals by transporting miRNAs and cytokines, contributing to disease progression and cardiovascular risks.	Investigating pEVs as therapeutic targets to manage inflammation and reduce cardiovascular risks in RA patients.	[106]
Atherothrombosis	pEVs carry stress-related proteins and modulate redox states, facilitating thrombus formation and platelet activation.	Targeting proteins carried by pEVs to prevent atherothrombosis progression; using hydroxychloroquine to reduce pEV-induced thrombosis risks.	[107,108]

## Data Availability

No new data were created or analyzed in this study. Data sharing is not applicable to this article.
